# Clinical imaging characteristics of inpatients with coronavirus disease-2019 in Heilongjiang Province, China: a retrospective study

**DOI:** 10.18632/aging.103633

**Published:** 2020-07-20

**Authors:** Hao Jiang, Wei Guo, Zhongxing Shi, Huijie Jiang, Mingyu Zhang, Lai Wei, Yongmei Pan

**Affiliations:** 1 Department of Radiology, The Second Affiliated Hospital of Harbin Medical University, Harbin, China; 2 Department of Ultrasound, Harbin The First Hospital, Harbin, China; 3 Department of Nuclear Medicine, Beijing Friendship Hospital, Affiliated to Capital Medical University, Beijing, China; 4 Department of Radiology, The First Affiliated Hospital of Harbin Medical University, Harbin, China; 5 Department of Radiology, Harbin Hong’an Hospital, Harbin, China

**Keywords:** COVID-19, SARS-CoV-2, pneumonia, severity of illness index, risk factors

## Abstract

Objective: To investigate the clinical, laboratory, and radiological characteristics of patients with coronavirus disease-2019 (COVID-19) in Heilongjiang Province.

Results: Patients in the ICU group were older and their incidence of cardiovascular disease was higher than those in the non-ICU group. Lymphocyte levels were lower and neutrophil and D-dimer levels were higher in the ICU than that in the non-ICU group. Compared to the non-ICU group, the incidence of pulmonary consolidation and ground-glass opacity with consolidation was significantly higher in the ICU group, all lung lobes were more likely to be involved, with higher number of lung lobes and areas surrounding the bronchi. Of the 59 patients with COVID-19 in this group, 15 received mechanical ventilation. All intubated patients involved lung lobes, and a large number of lesions were observed in the area around the bronchial vessels.

Conclusion: Significant differences were observed in clinical symptoms, laboratory tests, and computed tomography features between the ICU and non-ICU groups.

Methods: A total of 59 patients with COVID-19, comprising 44 patients in the intensive care unit (ICU) and 15 in the non-ICU, were retrospectively analyzed. Characteristics of the two groups of patients were compared.

## INTRODUCTION

An outbreak of new pneumonia caused by the 2019 novel coronavirus (2019-nCoV) started in Wuhan, China, in December 2019 [1]. In January 2020, Chinese scientists isolated this 2019-nCoV from patients with viral pneumonia, officially naming it as severe acute respiratory syndrome coronavirus 2 (SARS-CoV-2) [2]. Since then, the disease has rapidly spread from Wuhan to other regions. In February 2020, the World Health Organization (WHO) named the disease caused by this virus as coronavirus disease 2019 (COVID-19). At the time of this article's submission, some cases have been reported internationally across the six continents.

The COVID-19 pandemic has caused severe illness in infected patients, such as pneumonia and acute respiratory distress syndrome, which even resulted in death. According to the COVID-19 joint study report released by the National Health Commission of the People’s Republic of China, about 80% of patients have light and common infection, whereas 13.8% have severe/critical infections, making them highly at risk for mortality [3]. In addition, prevention and control of severe and critically ill patients are yet to be implemented [3]. Thus, clinicians and radiologists should identify the characteristic imaging manifestations in chest CT findings of critically ill individuals, so that they can perform specific symptomatic treatment at the earliest, prevent complications, and provide organ functional support. Compared to other methods, computed tomography (CT) is the best technique for the early detection of pneumonia. Only a few reports demonstrated the clinical imaging features of severe and critically ill patients during the epidemic in Heilongjiang Province. This study describes the clinical and radiological characteristics and laboratory examination data of 59 patients with COVID-19 and compares between those admitted in the intensive care unit (ICU) and non-ICU departments. Thus, we hope that these current results could be used by clinicians in Heilongjiang Province and worldwide for the treatment plan of COVID-19.

## RESULTS

A total of 59 patients confirmed with COVID-19 in Heilongjiang Province were included in this study. The general clinical data of patients are shown in Table 1. The median age was 64.0 (IQR, 56–72) years. The most common complication in the patient group was cardiovascular disease (44%), followed hypertension (42%) and diabetes (15%), and the rarest complication was chronic obstructive disease (3%), followed by malignancy (2%) and chronic liver disease (2%). Compared to non-ICU patients, ICU patients were older (median age: 67 *vs*. 56); *P* = 0.037) and more likely at risk for cardiovascular diseases (52% *vs*. 20%; *P* = 0.030). The most common clinical symptoms in this study were fever (41/59, 69%), cough (30/59, 51%), and muscle soreness (15/59, 25%), whereas the less common were dyspnea (14/59, 24%), headache (8/59, 13%), abdominal pain, diarrhea (5/59, 8%), and nausea (3/59, 5%). However, compared to non-ICU patients, the incidence of muscle soreness in the ICU patients was reduced (18% *vs*. 47%; *P* = 0.042).

**Table 1:** Demographics and baseline characteristics of two groups of patients infected with 2019-nCoV.

	**All patients (n=59)**	**ICU care (n=44)**	**No ICU care (n=15)**	***P* value**
**Characteristics**				
Age (y)	64.0(56.0-72.0)	66.5(57.3-75.8)	56.0(50.0-68.0)	0.037
Gender				0.552
Male	29(49%)	23(52%)	6(40%)	
Female	30(51%)	21(48%)	9(60%)	
Exposure history				0.516
Contact with infected patients	42(71%)	30(68%)	12(80%)	
Unknown history	17 (29%)	14(32%)	3(20%)	
Any comorbidity				
Diabetes	9(15%)	6(14%)	3(20%)	0.680
Hypertension	25(42%)	20(45%)	5(33%)	0.412
Cardiovascular disease	26(44%)	23(52%)	3(20%)	0.030
COPD	2(3%)	1(2%)	1(7%)	0.447
Malignancy	1(2%)	1(2%)	0(0%)	--
Chronic liver disease	1(2%)	0(0%)	1(7%)	--
**Signs and symptoms**				
Fever	41(69%)	31(70%)	10(67%)	0.785
Highest temperature, °C				0.412
<37.3	18(31%)	14(32%)	4(27%)	
37.3–38.0	25(42%)	16(36%)	9(60%)	
38.1–39.0	15(25%)	13(30%)	2(13%)	
>39.0	1(2%)	1(2%)	0(0%)	
Cough	30(51%)	20(45%)	10(67%)	0.205
Myalgia or fatigue	15(25%)	8(18%)	7(47%)	0.042
Headache	8 14%)	4(9%)	4(27%)	0.184
Diarrhoea, bellyache	5(8%)	4(9%)	1(7%)	0.624
Dyspnoea	14(24%)	9(20%)	5(33%)	0.316
Nausea	3(5%)	1(2%)	2(13%)	0.156

Data are median (IQR), n (%), or n/N (%), where N is the total number of patients with available data. *P* values comparing Group1 and Group2 are from χ² test, Fisher’s exact test, or Mann-Whitney U test. 2019-nCoV=2019 novel coronavirus. COPD=Chronic obstructive pulmonary disease.

Laboratory examination results of 59 patients are summarized in Table 2. White blood cell count (<4 × 10^9^/L; 11/59, 19%) and lymphocyte count (<1.0 ×10^9^/L; 26/59, 44%) were low in some patients. Compared to non-ICU patients, ICU patients are more likely to have lymphopenia (52% *vs*. 20%; *P* = 0.003), with higher neutrophil and D-dimer levels (median: 3.5 [IQR, 2.6–5.2] *vs*. median 1.7 [IQR, 0.8–3.1], *P* = 0.003; median 364.6 [IQR, 3.5–1475.0] *vs*. median 0.5 [IQR, 0.4–6.5], *P* = 0.000, respectively) and lower hemoglobin levels (median, 100.5 [IQR, 86.0–115.0] *vs*. median, 128.0 [IQR, 122.0–136.0], *P* < 0.001).

**Table 2:** Laboratory findings of two groups of patients infected with 2019-nCoV.

**Laboratory Findings**	**All patients (n=59)**	**ICU care (n=44)**	**No ICU care (n=15)**	***P* value**
White blood cell count(×10^9^/L)	5.5(4.3-7.1)	5.2(4.1-7.0)	5.8(4.6-7.0)	0.334
<4	11(19%)	9(20%)	2(13%)	0.894
4-10	42(71%)	30(68%)	12(80%)	
>10	6(10%)	5(11%)	1(7%)	
Ne utrophil count(×10^9^/L)	3.2(1.9-4.8)	3.5(2.6-5.2)	1.7(0.8-3.1)	0.003
Lymphocyte count(×10^9^/L)	1.1(0.6-1.5)	0.9(0.6-1.3)	1.6(0.9-2.3)	0.004
<1.0	26(44%)	23(52%)	3(20%)	0.030
≥1.0	33(56%)	21(48%)	12(80%)	
Haemoglobin, g/L	104.0(92.0-122.0)	100.5(86.0-115.0)	128.0(122.0-136.0)	0.000
Platelet count(×10^9^/L)	189.0(145.0-260.0)	194.5(142.0-264.5)	189.0(152.0-255.0)	0.734
<100	11(19%)	9(20%)	2(13%)	0.712
≥100	48(81%)	35(80%)	13(87%)	
Prothrombin time, s	12.4(12.0-13.3)	12.6(12.0-13.4)	12.0(11.9-13.0)	0.458
Activated partial thromboplastin time, s	30.9(28.0-33.3)	31.0(27.0-33.9)	30.5(29.0-31.8)	0.651
D-dimer, mg/L	6.1(1.5-1090.0)	364.6(3.5-1475.0)	0.5(0.4-6.5)	0.000
C-reactive protein, mg/L	8.4(2.0-30.9)	9.9(0.3-180.7)	8.0(0.2-77.9)	0.807
Alanine aminotransferase, U/L	37.6(30.2-45.0)	37.8(25.9-46.7)	36.7(34.4-40.7)	0.862
Aspartate aminotransferase, U/L	26.5(21.2-33.3)	26.5(19.3-35.0)	26.1(23.8-33.3)	0.708
≤40	51(86%)	36(82%)	15(100%)	0.100
>40	8(14%)	8(18%)	0(0%)	
Creatinine, μmol/L	57.1(44.7-89.9)	55.7(42.0-83.0)	89.9(57.0-133.0)	0.008
≤133	53(90%)	41(93%)	12(80%)	0.165
>133	6(10%)	3(7%)	3(20%)	
Creatine kinase, U/L	116.0(34.6-175.3)	130.1(34.8-200.0)	113.9(31.5-167.7)	0.676
≤185	45(76%)	32(73%)	13(87%)	0.483
>185	14(24%)	12(27%)	2(13%)	

Data are median (IQR) or n/N (%), where N is the total number of patients with available data. p values comparing Group1 and Group2 are from χ², Fisher’s exact test, or Mann-Whitney U test. 2019-nCoV=2019 novel coronavirus.

**Figure 1:**
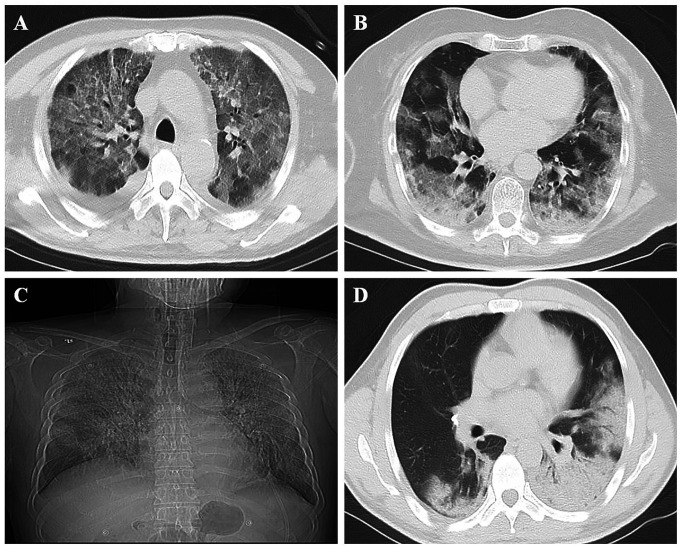
**Chest imaging of patients with COVID-19.** (**A**) Ground-glass opacity; (**B**) Lesion with ground-glass opacity and consolidation; (**C**) Lesion involving all lung lobes of both lungs; (**D**) Lesion involving the surrounding area of the bronchial blood vessel.

All patients (59/59; 100%) showed abnormal CT findings (Table 3). The main features of the imaging examination were ground-glass opacity (58/59; 98%; Figure 1A), consolidation (37/59; 63%), and ground-glass opacity combined with consolidation (36/59; 61%; Figure 1B). Compared to non-ICU patients, the incidence of consolidation and ground-glass opacity combined with consolidation in ICU patients was higher (73% *vs*. 33%, *P* = 0.006; 70% *vs*. 33%, *P* = 0.011, respectively). Furthermore, 40/59 (68%) patients showed involvement of all lung lobes in the ICU group (Figure 1C) as compared to the non-ICU patients, whereas the incidence of all lung lobes (75% *vs*. 47%, *P* = 0.043) and the number of lung lobes were higher in patients with ICU (median, 5 [IQR, 5–5] *vs*. median, 4 [IQR, 2–5], *P* = 0.012). Among 59 patients with COVID-19, 43 (73%) were multifocal, 15 (25%) were diffuse, and only 1 (2%) was focal. A significant difference was detected in the degree of lung involvement between ICU and non-ICU patients (*P* = 0.032). Furthermore, 23 (39%) patients had abnormal density shadows around the bronchi: 21/44 (48%) ICU patients and 2/15 (13%) non-ICU patients. The incidence of bronchovascular involvement in ICU patients was significantly higher than that in non-ICU patients (48% *vs*. 13%, *P* = 0.040), which might be observed by breathing difficulty and need for mechanical ventilation (Figure 1D). Unilateral or bilateral pleural effusion occurred in 7/59 (12%) patients: 6 in the ICU group (6/44, 14%) and 1 in the non-ICU group (1/15, 7%). In addition, mediastinal lymphadenopathy (short axis, >1 cm) was observed in 13 of 59 patients (22%), fibrous cord shadow in 22 (37%), and arterial plaque in 32 (54%).

**Table 3:** CT diagnosis characteristics of two groups of patients infected with 2019-nCoV.

**Imaging Findings**	**All patients (n=59)**	**ICU care (n=44)**	**No ICU care (n=15)**	***P* value**
Parenchymal opacities				
Consolidation	37(63%)	32(73%)	5(33%)	0.006
GGO	58(98%)	43(98%)	15(100%)	0.746
GGO and consolidation	36(61%)	31(70%)	5(33%)	0.011
Reticular opacities	13(22%)	7(16%)	6(40%)	0.073
Nodular opacities	11(19%)	8(18%)	3(20%)	0.574
Laterality				0.265
Bilateral	4(7%)	2(5%)	2(13%)	
Unilateral	55(93%)	42(95%)	13(87%)	
Involvement range of lung lobes				
All lung lobe	40(68%)	33(75%)	7(47%)	0.043
Right upper lobe	51(86%)	38(86%)	7(47%)	0.673
Right middle lobe	49(83%)	39(89%)	10(67%)	0.104
Right lower lobe	54(92%)	42(95%)	12(80%)	0.099
Left upper lobe	51(86%)	39(89%)	12(80%)	0.407
Left lower lobe	52(88%)	41(93%)	11(73%)	0.062
Number of lung lobes, mean	5(4-5)	5(5-5)	4(2-5)	0.012
Distribution				
Central and peripheral	9(15%)	8(18%)	1(7%)	0.424
Central	12(20%)	11(25%)	1(7%)	0.160
Peripheral	53(90%)	39(89%)	14(93%)	0.518
Peribronchovascular	23(39%)	21(48%)	2(13%)	0.040
Extent				0.032
Single shot	1(2%)	0(0%)	1(7%)	
Multiple	43(73%)	30(68%)	13(87%)	
Diffuse	15(25%)	14(32%)	1(7%)	
Pleural effusion	6(10%)	3(7%)	3(20%)	0.165
Arterial plaque	22(37%)	15(34%)	7(47%)	0.384
Fiber rope	32(54%)	27(61%)	5(33%)	0.060
Mediastinal lymphadenopathy	13(22%)	10(23%)	3(20%)	0.569

Data is n/N (%), where N is the total number of patients with available data. Abbreviations: CT, computed tomography; GGO, ground-glass opacity.

A total of 15 (25%) patients were intubated with respiratory failure. All of them (100%) had ground-glass opacity, showed bilateral lung involvement, and involved more than three lung lobes. Compared to the non-mechanically ventilated patients, these patients requiring mechanical ventilation were more likely to have abnormal lung changes in the area around the bronchi (53% *vs*. 34%) and showed diffuse distribution (47% *vs*. 18%).

## DISCUSSION

COVID-19 is a new viral outbreak that may have a profound impact on public health. With the increased number of confirmed cases, the number of severe and critical cases in Heilongjiang Province is also continuously increasing. This might be caused by lung tissue inflammation, which in turn, causes organ dysfunction and is even life-threatening. In addition, patients who are severely/critically ill have poor prognosis and higher mortality than non-critically ill ones [6, 7]. A recent assessment showed that the fatality rate of severe pneumonia is 30–50%, leading to severe complications and increasing the medical burden [8]. Thus, early identification of such cases based on changes in chest radiography and clinical features is crucial. In the present study, clinical and imaging characteristics of patients with COVID-19 in the ICU group were determined by comparing the ICU and non-ICU patients.

The most common clinical symptoms in this group of patients were fever and cough. We found that the ICU group was older and more likely to have cardiovascular disease than the non-ICU group. Moreover, older people or people with poor health conditions were found to have a worsening pneumonia, which might be due to the weakened immune system [9]. According to a study report on patients with COVID 19 in Wuhan [10], the probability of all patients with hypertension and cardiovascular disease is 15% and 15%, whereas the corresponding incidence in patients with COVID 19 in Heilongjiang Province is 42% and 44%, which may be attributed to the specific geographical environment of Heilongjiang Province, resulting in a high incidence of cardiovascular diseases. Studies on SARS-CoV and Middle East Respiratory Syndrome (MERS)-CoV infections demonstrated that the risk of exacerbation markedly increases with age and presence of underlying diseases [11–13], which was consistent with the conclusions of this study. The difference in the male-to-female ratio was not significant between the two groups, indicating that gender is not a high-risk cause of disease severity, which is consistent with that of a recent report [14]. Compared to the ICU group, the incidence of muscle soreness was significantly higher in the non-ICU group. This clinical symptom is rarely observed in other related studies and may be related to regional environmental characteristics. Taken together, these clinical manifestations can help clinicians determine the disease severity in clinical practice. Other symptoms in our patients with COVID-19 were similar to that of other coronavirus infections, including dyspnea, headache, abdominal pain, diarrhea, and nausea. For example, SARS and MERS may belong to the same attributed infection and also indicate that the SARS-CoV-2 target cells are located in the lower respiratory tract [15–17].

The present study identified multiple laboratory index differences between non-ICU groups and ICU groups, including lymphocyte, neutrophil, and D-dimer levels. Compared to the non-ICU group, the ICU group is prone to lymphopenia, which is consistent with the results of the latest research report of patients with COVID-19 in Wuhan and China [10, 18]. Lymphopenia in the ICU group indicates that a large number of immune cells are consumed and the immune function is suppressed, demonstrating that lymphocyte damage may be the key to the deterioration of the patient’s condition; therefore, decreased lymphocyte count could be a critical indicator of disease severity [19]. Increased neutrophil and D-dimer levels in patients in the ICU group may be related to cytokine storms caused by the viral invasion, which is supported by recent studies [9, 20]. Notably, patients with high D-dimer levels for the first time are predictive of poor prognosis [20], which is consistent with the opinion of this study.

From a broad perspective, CT manifestations of COVID-19 pneumonia are similar to that of other viral pneumonia. Imaging findings of viral pneumonia include reticular pattern and patchy or diffuse ground-glass opacity, with or without consolidation [21]. In influenza pneumonia, lobular septal thickening and grid-like density shadows are frequently observed, whereas pleural effusion is rare [21]. Despite similarities, some of our patients’ imaging findings are different from those of the traditional seasonal flu.

In this study, all patients with COVID-19 had abnormal chest CT findings. Additionally, ground-glass opacity (98%) and consolidation (63%) are the most common imaging findings in the current study, which is consistent with the results of the recent COVID-19 studies [22]. This phenomenon may be related to exudative inflammation caused by alveolar and interstitial edema of the lung due to viral invasion, and CT is mainly manifested as ground-glass opacity [23]. An autopsy report of patients with COVID-19 pneumonia deaths shows that the ground-glass opacity corresponds to the gray-white alveolar lesions observed by the naked eye, suggesting that the virus mainly causes inflammatory reactions characterized by deep airway and alveolar damage [24]. Herein, we found that compared to the non-ICU group, the incidence of consolidation and ground-glass opacity combined with consolidation in patients in the ICU group was higher (*P* = 0.006; *P* = 0.011), indicating that the alveoli of critically ill patients were filled with inflammatory exudates. This means that the virus has spread to the respiratory tract, leading to necrotic bronchitis and diffuse alveolar damage [25, 26], which is consistent with the results of recently published studies [27–29]. Among the 59 (68%) patients, 40 displayed imaging abnormalities involving all lung lobes (5) as compared to 7/15 (47%) of non-ICU patients, whereas 33/44 (75%) of all ICU patients were involved; the difference between the two groups was statistically significant (*P* = 0.043). In addition, we found that the degree of involvement of lung lesions was statistically significant between the two groups (*P* = 0.032). Chest imaging features may help the early prediction of the patients’ clinical development early.

In this group of patients, 15 needed mechanical ventilation. Compared to non-mechanical ventilation patients, CT abnormalities in the lungs of patients requiring mechanical ventilation were primarily distributed around the bronchial blood vessels, and diffuse distribution was likely to occur, making patients prone to dyspnea. Some other studies demonstrated that the distribution of abnormal lesions during CT examination may be the decisive factor for the clinical course of patients with COVID-19 [22, 30]. Other imaging features in this study included bilateral lung involvement in 93% of patients, and majority of them (90%) had lung lesions in the peripheral area without emphysema or pulmonary nodules; these imaging abnormalities and distribution patterns are consistent the previously published results [31, 32]. Among the patients in this study, only 7 (12%) had pleural effusion, including 6 (14%) in the ICU group and 1 (7%) in the non-ICU group. Furthermore, pleural effusion is a rare imaging manifestation in patients with COVID-19, and the incidence rate in the ICU group is higher than that in the non-ICU group, which is consistent with the results of Junhua et al.’s study [33].

Nevertheless, this study has some limitations. (1) None of the patients underwent lung biopsy or autopsy, which might have established a correlation between imaging and histopathology. (2) The sample size of the non-ICU group is relatively small. Collecting standardized data for larger populations will help explore clinical manifestations and high-risk factors. (3) As most patients are still in the hospital at the time of submission of this manuscript, risk factors for poor prognosis were not assessed.

## CONCLUSIONS

In summary, existing cardiovascular disease, fever, and cough in elderly patients with COVID-19 may worsen the condition. Lymphopenia and elevated neutrophil and D-dimer levels are also indicators of COVID-19 disease progression. In addition, imaging findings of patients with severe COVID-19 mainly include consolidation and ground-glass opacity combined with consolidation, which putatively involves all lung lobes and the area around the bronchi. Since several patients are currently in the critical stage, we hope that the results of this study would be beneficial for the disease control, diagnosis, treatment, and prognosis in Heilongjiang Province and worldwide and even reduce the mortality rate.

## MATERIALS AND METHODS

### Study population

The study has been approved by the Ethics Committee of the Second Affiliated Hospital of Harbin Medical University and is in accordance with the Helsinki Declaration. According to the COVID-19 pneumonia diagnostic criteria for the diagnosis and treatment of new coronavirus-caused pneumonia (trial version 6) issued by the National Health Commission of the People’s Republic of China [4], the inclusion criteria were as follows: (1) real-time fluorescent reverse transcription-polymerase chain reaction (RT-PCR) for detection of positive cDNA of SARS-CoV-2; (2) untreated newly diagnosed patients; (3) patients with complete clinical data; and (4) all patients who underwent at least one CT scan. Exclusion criteria were as follows: (1) treated non-newly diagnosed patients and (2) missing clinical data. This study included a total of 76 patients confirmed with COVID-19 between February and March 2020, and 59 of them met the above criteria. The cohort was divided into the ICU (n = 44) and non-ICU groups (n = 15). Clinical data of all patients were evaluated: background information such as gender and age and clinical symptoms such as fever, cough, and underlying diseases (hypertension, diabetes, cardiovascular disease, and chronic obstructive pulmonary disease). Laboratory examination results upon admission, including white blood cells, lymphocytes, neutrophils, D-dimer, and C-reactive protein levels, as well as imaging data, were collected.

### Image analysis

All CT images were analyzed and diagnosed by two radiologists trained for novel coronavirus. Both radiologists have >5 years of diagnostic experience. Two doctors independently diagnosed all patient images and reached a consensus. In case of disagreement between the two radiologists, a third trained radiologist with >10 years of diagnostic experience was consulted to reach a consensus. Imaging features (ground-glass opacity, consolidation, reticular pattern, and nodular opacity), lesion distribution (unilateral/bilateral, upper/middle/lower lobe, and central/peripheral/bronchial blood vessel surrounding), and degree of involvement (focal/multifocal/diffuse and number of lung lobes) were all abnormal. Radiographic images and CT scans using descriptors were defined using the Fleischner Society Naming Committee [5]. Ground-glass opacity is defined as a hazy area showing increased lung opacity with indistinct pulmonary vessel margins on a radiograph but with preserved bronchial and vascular margins on CT. Consolidation is defined as a homogeneous increase in parenchymal attenuation that obscures vessel margins and airway walls. The reticular pattern is defined as small linear opacities forming a net pattern. Nodular opacity is defined as a well- or poorly defined rounded opacity, measuring up to 3 cm in diameter. Lesion distribution features include unilateral/bilateral and upper/middle/lower lobes. The extent of lesion involvement was divided into focality, multifocality, and diffuse. Focality is defined as an abnormal single lesion, whereas multifocality is defined as the presence of more than one lesions; if it is diffusely distributed, it involves one or both lungs. Moreover, whether the lesion occurs centrally (<4 cm from the hilum) or peripherally or involves the bronchi should be determined. The presence of pleural effusion, laterality, and any other lung findings such as mediastinal lymphadenopathy was also noted.

### Statistical analysis

The SPSS 19.0 statistical software was used for analysis. Continuous variables were expressed as median (interquartile ratio [IQR]) and compared using the Mann–Whitney U test. Categorical variables were expressed as number of cases (n) and percentage/rate (%); χ² test or Fisher’s exact test was used to compare ICU and non-ICU groups. *P* < 0.05 was considered statistically significant.
